# Two Case Reports of Patients With Transverse Myelitis as a Complication of SARS-CoV-2 Infection

**DOI:** 10.7759/cureus.29191

**Published:** 2022-09-15

**Authors:** Valentina Maksymyuk, Sarah Doell, Natallia Maroz

**Affiliations:** 1 Internal Medicine, Kettering Medical Center, Dayton, USA; 2 Nephrology, Wright State University, Dayton, USA

**Keywords:** sars-cov-2 infection, sars-cov-2, plasmapheresis, longitudinally extensive transverse myelitis, acute transverse myelitis (atm), transverse myelitis

## Abstract

Transverse myelitis is a nontraumatic spinal cord injury that presents with sudden onset weakness, sensory deficits, and autonomic dysfunction. It can be caused by multiple etiologies including malignancy, autoimmune disorders, viral, bacterial, or fungal infections, and environmental factors. In this article, we describe cases of two elderly male patients affected by the SARS-CoV-2 virus. Patients did not exhibit classic or had only mild classic symptoms of SARS-CoV-2 infection; however, both patients developed transverse myelitis. Patients were treated with intravenous steroids and therapeutic plasmapheresis, achieving partial improvement. The study aimed to understand rare complications like transverse myelitis of SARS-CoV-2 infection and treatment accordingly.

## Introduction

Classic presentations of SARS-CoV-2 infection experienced by the majority of infected individuals are similar to the common cold, including fever, chills, body aches, loss of taste and smell, nasal congestion, and dry cough [1]. However, from the beginning of the pandemic, it was clear that SARS-CoV-2 affects multiple organ systems. Neurological manifestations from SARS-CoV-2 infection range from metabolic encephalopathy, headaches, confusion, and dizziness to more serious complications including thromboembolic stroke [2], transverse myelitis (TM) [3,4], Guillain-Barré syndrome [5], encephalitis, and meningitis [6]. This is our contribution to the case reports of SARS-CoV-2 infection leading to acute TM. We present two cases of male patients who were treated for acute TM caused by SARS-CoV-2 infection with therapeutic plasmapheresis.

## Case presentation

Case 1

A 68-year-old man, unvaccinated against SARS-CoV-2, presented to the hospital with acute onset of lower extremity weakness. His past medical history was significant for congestive heart failure with preserved ejection fraction, atherosclerotic heart disease, chronic obstructive pulmonary disease, and subdural hematoma secondary to a motor vehicle collision in 2020.

He was in his regular state of health until five days prior to hospitalization in March 2021. He was a fully functional male for his age and was living independently, ambulating, and able to do low-intensity exercises such as weight lifting. His initial symptoms were bilateral lower extremity weakness, which quickly progressed to complete paraplegia with loss of sensation and complete loss of strength in the left upper extremity. He was unable to lift himself from a chair or ambulate. Lastly, he developed urinary retention.

Upon arrival at the emergency department, a review of systems was negative for classic symptoms of SARS-CoV-2, including fevers, chills, shortness of breath, cough, fatigue, muscle aches, abdominal discomfort, or diarrhea. Vital signs were within normal limits with oxygen saturation of 100% on ambient air. The physical exam revealed the patient was alert and oriented; his thought process was logical, coherent, and linear; he was able to provide an accurate history. Strength was 0/5 in the left upper and lower extremities, 1/5 in the right lower extremity, and 5/5 in the right upper extremity. Patellar reflexes and biceps tendon reflexes were 1/4; Achilles tendon reflexes were absent. The patient had reduced sensation below the sternal notch with significantly diminished sensation to light touch, temperature, pinprick, and vibration in the lower extremities, but all were intact in the upper extremities. The American Spinal Injury Association (ASIA) score was A.

**Figure 1:**
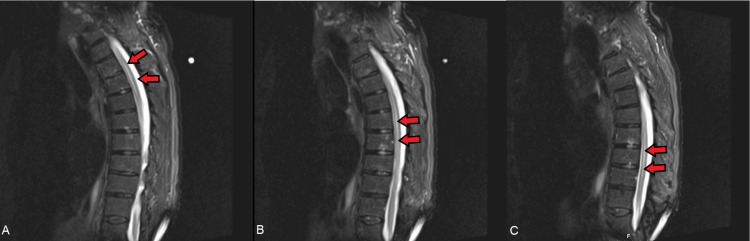
MRI of the thoracic spine (Patient #1). (A) Upper thoracic spine. (B) Middle/lower thoracic spine. (C) Lower thoracic spine. There is diffuse central T2 lengthening throughout the thoracic cord at all segments consistent with transverse myelitis.

The timing of his hospitalization coincided with a peak in the SARS-CoV-2 pandemic; therefore, the SARS-CoV-2 polymerase chain reaction (PCR) test from nasopharyngeal secretion was collected and reported as positive. Other laboratory tests were significant for elevated C-reactive peptide at 33.70 mg/L, D-dimer at 500 ng/mL (0-241 ng/mL), mild leukocytosis with neutrophilic predominance at 13.4 K/uL (4.0-10.5 K/uL), mild normocytic anemia Hg at 12.3 (13.1-17.6), hematocrit (Hct) at 36.7 (39.0-51.5), and thrombocytosis at 541 K/uL (154-393 K/uL). The MRI of the C1-T2 spinal level was suggestive of TM in addition to severe degenerative changes in the cervical spine region and possible cord compression (Figure 1).

Since the differential diagnosis included spinal cord compression, the patient was promptly evaluated by neurosurgery for acute compression of the spinal cord, which was ruled out. Electromyography (EMG) was performed, and results were not consistent with acute inflammatory demyelinating polyneuropathy (AIDP) or other demyelinating disorders; however, there was evidence of mild peripheral polyneuropathy as most sensory responses were small or absent. Lumbar puncture returned clear colorless cerebrospinal fluid (CSF) benign for infectious processes with normal white blood cells (9 cell/uL), red blood cells (98 cells/uL), granulocytes (2 cell/uL), elevated fluid lymphocytes at 95% (40-80%), elevated fluid protein at 74 mg/dL (15-45 mg/dL), elevated fluid glucose at 91 mg/dL (40-70 mg/dL), which was normal compared to serum glucose of 211 mg/dL, and elevated myelin basic protein (0.0-5.4 ng/mL). The fluid culture showed white blood cells, but no organisms were seen, and the autoimmune workup was unrevealing: NMO-AQ4-IGG were <1:1. Blood and urine cultures showed no growth, and varicella-zoster PCR was negative.

Based on the available data, the patient was diagnosed with acute TM presumed secondary to SARS-CoV-2 infection. Treatment with intravenous infusion of methylprednisolone 1000 mg once daily for five days was initiated. Management of urinary retention required placement of a Foley catheter.

Unfortunately, completion of the course of methylprednisolone therapy had very little impact on the patient’s condition, and a significant neurological deficit remained. Therapeutic plasma exchange (TPE) was considered the next step of clinical management. In accordance with the American Society for Apheresis (ASFA) recommendation, the patient underwent the course of five treatments using a 5% albumin solution as a replacement fluid. Remarkably, the patient’s muscular strength had significantly improved upon completion of the first TPE session. At the end of the course of five treatments, he was able to lift both of his lower extremities against gravity. The ASIA grade improved from A to D.

A voiding trial was performed before discharge, and the patient was able to urinate adequately on his own. The patient was evaluated and treated by physical and occupational therapy with recommendations for facility-based therapy services. He was discharged to the skilled nursing facility placement for further rehabilitation of physical strength and independence.

Case 2

A 64-year-old man, unvaccinated against SARS-CoV-2, was transferred to our facility from the outside hospital with an already working diagnosis of acute TM in the setting of SARS-CoV-2 infection for treatment with TPE. His past medical history included polymyalgia rheumatica treated with prednisone, nephrolithiasis, benign prostatic hyperplasia complicated by intermittent episodes of urinary retention, and a small infarct of the left cerebral hemisphere in 2011 with no residual deficit.

At the beginning of October 2021, the patient experienced symptoms of headaches, fevers, chills, diarrhea, and decreased appetite. The initial rapid SARS-CoV-2 test was negative, and symptoms improved with supportive measures at home. However, two weeks later, the patient developed a new onset of fatigue, generalized weakness, and subtle numbness in both feet. In the next three days, numbness associated with weakness involved both lower extremities and a portion of the torso below the nipple. He could not get himself up to stand and developed difficulties with urination and bowel movements.

He presented to the emergency room, where he was noticed to be hemodynamically stable, saturating 100% on ambient air, and was afebrile. The physical exam revealed the patient was alert and oriented to the situation, time, and place, his speech was clear and language fluent, and he was following commands appropriately. His strength was 5/5 in the upper extremities bilaterally, 1/5 in the lower extremity, and 2/5 in the right lower extremity. Patellar reflexes and biceps tendon reflexes were 1/4. Deep tendon reflexes were 2/4 in the biceps, triceps, brachioradialis, patellar, and Achilles bilateral; flexor plantar responses were bilateral, and no pathological reflexes were appreciated. The patient had an intact light sensation to touch in upper and lower extremities bilaterally; however, he had decreased pinprick sensation below T4. Since he had some sensory and partial motor function preserved below the neurological level, the initial ASIA grade was C.

**Figure 2:**
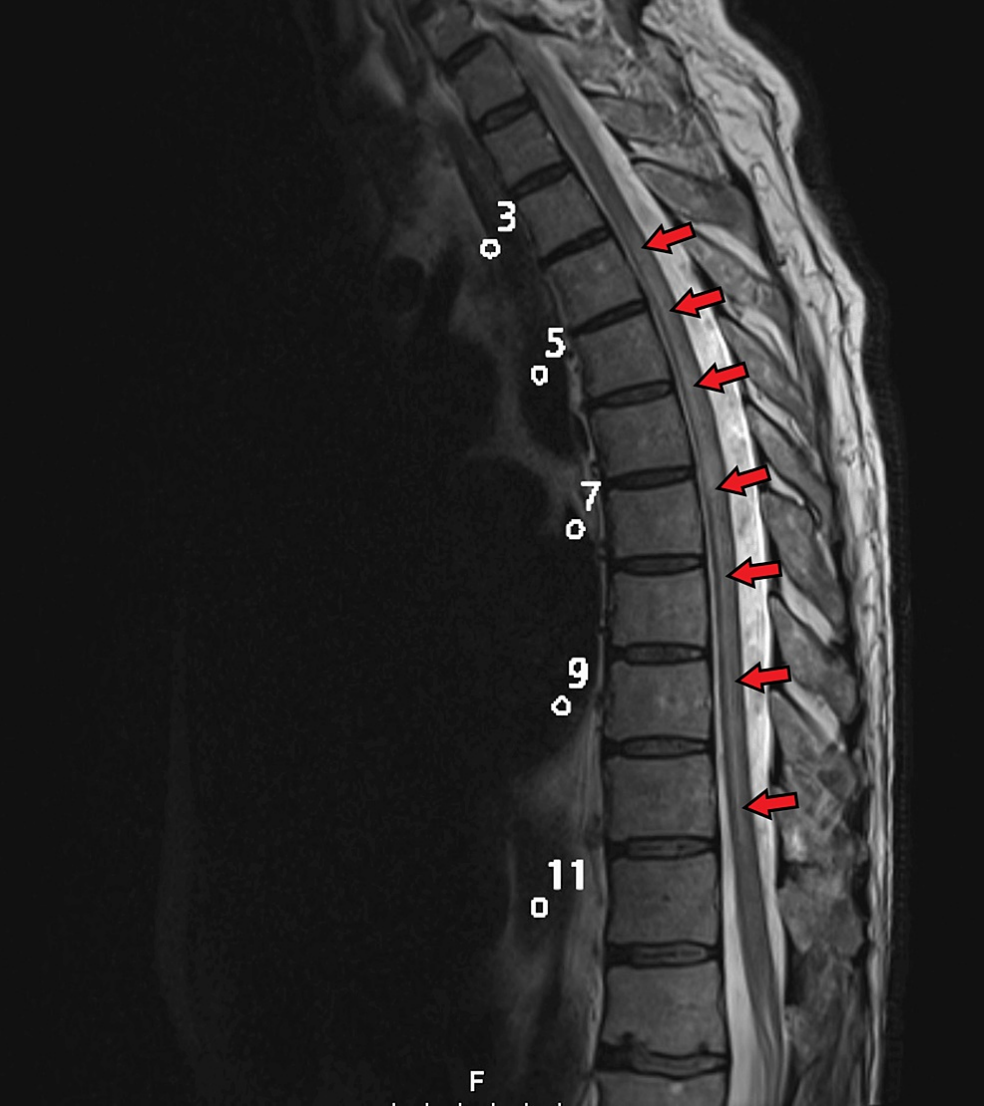
MRI of the thoracic spine (Patient #2). A long segment of high signal in the spinal cord from T3-T4 through T10-T11 without associated enhancement; findings are concerning for transverse myelitis.

Repeat nasopharyngeal rapid SARS-CoV-2 test was positive. Other laboratory tests were significant for hypokalemia (2.9 mmol/L) and hypomagnesemia (1.8 mg/dL), and urinalysis with ketonuria (20 mg/mL). Complete blood count did not reveal leukocytosis (4.8 K/uL) (range: 4.0-10.5 K/uL), but showed mild normocytic anemia Hg at 12.7 (13.1-17.6), Hct at 36.7 (39.0-51.5), and thrombocytosis at 479 K/uL (154-393 K/uL). Lumbar puncture returned clear colorless CSF fluid indicative for inflammatory process with elevated white blood cells count at 227 cell/uL with granulocytes at 87% (≤25%), red blood cells at 36 cells/uL, lymphocytes at 7% (≤75%), monocytes at 6% (≤70%), elevated protein at 63 mg/dL (15-45 mg/dL), and elevated fluid glucose at 73 mg/dL (40-70 mg/dL), which was normal compared to serum glucose of 196 mg/dL. CSF PCR panel for infectious etiology including *Escherichia coli*, *Haemophilus influenzae*, *Listeria monocytogenes*, *Streptococcus agalactiae*, *Streptococcus pneumoniae*, *Cytomegalovirus*, human herpesvirus 6, human parechovirus, varicella-zoster virus, enterovirus, herpes simplex virus 1 and 2, and *Cryptococcus neoformans*/*Cryptococcus gattii* was negative. Autoimmune workup was unrevealing: NMO-AQ4-IGG were <1:1; oligoclonal banding and angiotensin-converting enzyme CSF were negative. Myelin basic protein was elevated at 167 ng/ml (0.0-5.4 nh/mL), which is nonspecific and only indicative of myelin breakdown. CSF flow cytometry was negative with no immunophenotypic evidence of monotypic B-cell or aberrant T-cell population. Cytology was negative for malignancy. Blood and urine cultures were negative. The MRI of cervical, thoracic, and lumbar spinal levels with and without contrast was performed. MRI of the cervical spine and lumbar spine showed some degenerative changes, but no acute pathology. MRI of the thoracic spine revealed a long segment of high signal in the spinal cord from T3-T4 through T10-T11 (Figure 2).

Based on the clinical picture, laboratory workup, and imaging studies, acute TM with SARS-CoV-2 post-infectious etiology was suspected. Treatment with intravenous infusion of methylprednisolone 1000 mg once daily for five days was initiated. A Foley catheter was placed for the management of urinary retention.

As in the first case, completion of the course of methylprednisolone therapy had only minimal improvement on the patient’s condition and a significant neurological deficit remained. At this time, the patient was transferred to our facility for initiation of TPE. The patient underwent the course of nine treatments using a 5% albumin solution as a replacement fluid. The patient's muscular strength significantly improved upon completion of the first TPE session. At the end of the course of five treatments, he was able to lift both of his lower extremities against gravity. ASIA score improved from C to D.

The patient was evaluated and treated by physical and occupational therapy with recommendations for facility-based therapy services. The patient was able to restore regular normal bowel movements regimen with MiraLAX and Dulcolax suppositories; unfortunately, he failed two voiding trials and a Foley catheter had to be continued with eventual follow-up with urology.

## Discussion

TM is a rare neurological disease in which heterogeneous inflammatory processes affect a portion of the spinal cord [7]. A multitude of processes has been implicated in the etiology of TM, including infection, malignancy, environmental toxins, medications, autoimmune disease, or demyelinating diseases such as multiple sclerosis [7]. Hence, obtaining a comprehensive medical, social, family, travel, and vaccination history is of paramount importance.

Physical examination should include a thorough neurological assessment. TM can present in a variety of ways depending on the anatomical location of the spinal tract involved. The differential diagnosis is broad and involves other spinal cord disorders, such as anterior spinal cord syndrome, central cord syndrome as a complication of the syrinx, compression of the spinal cord, Brown-Sequard syndrome, spinal abscess, cord infarction, and herniated disc, as these abnormalities may present with overlapping symptoms [8]. MRI with gadolinium contrast is the gold standard for the diagnosis of TM and will help rule out other etiologies [7,8]. In the first case reported, neurosurgery was involved to evaluate for cord compression in the setting of the known history of cervical spine stenosis. In the second case reported, an MRI of the thoracic spine revealed a long segment of high signal in the spinal cord from T3-T4 through T10-T11 (Figure 2), correlating with findings of a longitudinally extensive TM, which is a rare entity since TM more commonly involves only a short segment of the spinal cord. Longitudinally extensive TM is associated with devastating outcomes.

The use of intravenous steroids is generally accepted as a first step in the treatment of TM. Although no randomized clinical studies have been completed, the use of intravenous methylprednisolone with and without taper has been reported to alleviate symptoms and decrease the number of days until full recovery. TPE is another treatment modality utilized for patients with moderate to severe TM with autonomic and motor impairment or for patients who did not significantly improve with the use of steroids [8]. The success of TPE in the case of viral etiology of TM has been reported previously but not specifically for the SARS-CoV-2 virus. In the first and second cases, patients had only a mild improvement in strength after steroid therapy but a robust response to TPE treatment has been observed, resulting in near regaining of neurologic function.

For long-term management and rehabilitation, work with physical and occupational therapies is very important. Prevention of skin pressure ulcers due to prolonged immobility along with increasing strength and flexibility of affected group muscles are two main goals. Consideration of managing bladder and bowel functions is also crucial in the recovery phase. It is very important to educate families and friends about the expected disease course since patients who have been independent before the onset of illness might have lengthy recovery and severely diminished physical performance and therefore require support and care from their loved ones [9].

Several theories have been proposed as to why the SARS-CoV-2 virus can present with nervous system abnormalities. The first theory is that the virus has a tropism to angiotensin-converting enzyme 2 receptors, and it directly invades nervous system cells (glial cells and neurons) through these receptors exactly the same way it invades the epithelium of the lung and gastrointestinal tract and vascular endothelium [10]. More research is needed to learn why the same virus may have an affinity to the nervous system in the absence of other symptoms as in case 1 presented in this article. The second theory also describes a direct invasion of the virus to the central nervous system through the olfactory bulb [11], which could explain why many patients have experienced a loss of smell and taste [12]. A third theory describes a path in which the virus can indirectly affect the nervous system through a cytokine storm, which causes inflammation mediated mostly by interleukins 6 and 8 as well as chemoattractant protein-1. Inflammation increases the permeability of the blood-brain barrier, which allows the passage of more inflammatory cytokines and chemokines, which can lead to subsequent damage [13]. Hypoxia, which is a common effect of SARS-CoV-2 infection, can lead to nervous system damage [14]. In addition to these proposed mechanisms, it should also be noted that certain treatments being used for severe SARS-CoV-2 infections can also cause nervous system dysfunction. For example, steroids, broad-spectrum antibiotics, prolonged sedation, and the use of paralytic agents in prone patients are heavy contributors to nervous system dysfunction.

## Conclusions

As cases of SARS-CoV-2 continue to rise across the globe, knowing different presentations of the disease will aid in both the diagnosis and treatment of patients. SARS-CoV-2 usually presents classic symptoms similar to the common cold and can progress to severe viral pneumonia. However, there are some unusual presentations of SARS-CoV-2 infection, which include neurological diseases. We described here two cases of patients who were infected by SARS-CoV-2 and did not present with classic symptoms of SARS-CoV-2 pneumonia. Instead, both developed acute lower extremity deficits associated with sensory loss and urinary retention and were diagnosed with acute TM. Patients were successfully treated with IV steroids and plasmapheresis. Both patients presented in this article were unvaccinated against SARS-CoV-2.
